# The Role of Antioxidants in the Management of Polycystic Ovary Syndrome

**DOI:** 10.3390/antiox15040487

**Published:** 2026-04-14

**Authors:** Tamara Sorić, Marijana Matek Sarić, Snježana Herceg Romanić, Ana Sarić, Antonija Jonjić, Miran Čoklo

**Affiliations:** 1 Psychiatric Hospital Ugljan, Otočkih dragovoljaca 42, 23275 Ugljan, Croatia; 2 Department of Health Studies, University of Zadar, Splitska 1, 23000 Zadar, Croatia; marsaric@unizd.hr; 3 Institute for Medical Research and Occupational Health, Ksaverska cesta 2, P.O. Box 291, 10001 Zagreb, Croatia; sherceg@imi.hr; 4 School of Medicine, Catholic University of Croatia, Ilica 242, 10000 Zagreb, Croatia; asaric1@unicath.hr; 5 Institute for Anthropological Research, Ljudevita Gaja 32, 10000 Zagreb, Croatia; antonija.jonjic@inantro.hr (A.J.); mcoklo@inantro.hr (M.Č.)

**Keywords:** polycystic ovary syndrome, oxidative stress, redox signaling, antioxidants, insulin resistance, mitochondrial dysfunction, gut microbiota, clinical outcomes

## Abstract

Polycystic ovary syndrome (PCOS) is a heterogeneous endocrine–metabolic disorder characterized by endocrine disruption, insulin resistance, hyperandrogenism, and chronic low-grade inflammation, in which oxidative stress has been proposed as a mechanistic link between metabolic and reproductive dysfunction. This narrative review summarizes current evidence on redox-related mechanisms and evaluates dietary and supplemental antioxidants in PCOS. Clinical trials, systematic reviews, and mechanistic studies were examined to assess antioxidant classification, signaling pathways, and outcomes related to metabolic, endocrine, reproductive, and oxidative stress parameters. Antioxidant interventions frequently modify circulating redox biomarkers and may improve selected metabolic indices; however, consistent effects on hormonal regulation, ovulation, and long-term clinical outcomes remain limited and heterogeneous. Differences in study design, antioxidant formulation and dosage, baseline metabolic status, and outcome selection complicate interpretation, while emerging evidence suggests modulation by lifestyle factors and gut microbiota-related mechanisms. Overall, antioxidants appear to act primarily through modulation of endogenous redox regulation rather than direct reactive oxygen species scavenging and are best considered adjuncts to lifestyle-based management. Further phenotype-informed and longitudinal studies using clinically relevant endpoints are required to clarify therapeutic relevance in PCOS.

## 1. Introduction

Polycystic ovary syndrome (PCOS) is among the most common endocrine disorders affecting women of reproductive age, with a global prevalence estimated to range between 8% and 20%, although reported rates vary considerably according to the diagnostic criteria applied [1,2,3,4,5]. Substantial regional variation has been reported. For example, in India, prevalence estimates range from 3.7% to 22.5%, reflecting both population heterogeneity and differences in diagnostic approaches [6]. The use of multiple diagnostic frameworks, together with the broad spectrum of clinical manifestations, continues to complicate epidemiological assessment and comparison across studies [4].

Clinically, PCOS is characterized by hyperandrogenism, chronic anovulation, and polycystic ovarian morphology [7,8]. These features are commonly accompanied by hirsutism, acne, menstrual irregularities, infertility, and a wide range of metabolic disturbances [9]. Beyond its reproductive manifestations, PCOS is closely linked with a range of metabolic disturbances, including endocrine disruption, insulin resistance, type 2 diabetes mellitus, obesity, dyslipidemia, and metabolic syndrome, all of which contribute to elevated long-term cardiovascular risk [10]. Although the syndrome is most often identified during the reproductive years, accumulating evidence suggests that these metabolic and cardiovascular consequences frequently persist later in life, highlighting the importance of long-term monitoring and risk management in affected women [1,10].

The pathophysiology of PCOS is widely recognized as multifactorial. Several interacting processes are involved, including endocrine disruption—abnormalities in ovarian steroid production and disruption of hypothalamic–pituitary–ovarian axis regulation, insulin resistance accompanied by compensatory hyperinsulinemia, and a state of chronic low-grade inflammation [10,11,12]. According to the Rotterdam diagnostic criteria, PCOS is diagnosed when at least two of the following features are present: clinical or biochemical hyperandrogenism, oligo- or anovulation, and polycystic ovarian morphology detected by ultrasound [1].

Oxidative stress (OS) has been increasingly discussed as a mechanism linking the metabolic, inflammatory, and reproductive components of PCOS. OS develops when the generation of reactive oxygen species (ROS) surpasses the capacity of endogenous antioxidant defenses, leading to oxidative modification of lipids, proteins, and nucleic acids. Elevated ROS levels have been associated with disturbances in follicular development, reduced oocyte competence, ovulatory dysfunction, and altered steroidogenic activity [10,11,12]. In addition, insulin resistance and androgen excess may further intensify oxidative burden through mechanisms involving mitochondrial dysfunction, increased inflammatory signaling, and endothelial impairment, thereby reinforcing a cycle of endocrine disruption, metabolic and reproductive dysregulation [13].

Importantly, ROS are not solely harmful by-products of metabolism. At physiological levels, they participate in normal cellular signaling processes related to metabolism, inflammatory responses, and ovarian physiology [12,14]. For this reason, disruption of the balance between oxidant production and antioxidant defense—rather than ROS accumulation alone—may be particularly relevant to understanding the role of redox disturbances in PCOS [12,14].

Given this proposed role of OS in endocrine disruption, linking metabolic and reproductive dysfunction, antioxidant-based interventions have attracted interest as adjunctive therapeutic strategies. Antioxidants, produced endogenously or obtained through diet and supplementation, may neutralize excessive ROS, modulate redox-sensitive signaling pathways, and limit oxidative damage. Experimental and clinical studies suggest that antioxidant interventions may improve insulin sensitivity, reduce androgen excess, support ovarian function, and attenuate inflammatory processes in women with PCOS [12,15]. However, the application of antioxidant therapy in clinical practice remains limited by heterogeneity in study design, variability in antioxidant type and dosage, short intervention durations, and lack of standardized outcome measures [1,12].

Emerging evidence also suggests that responses to antioxidant interventions vary across PCOS phenotypes and may be influenced by obesity status, degree of insulin resistance, hyperandrogenism, and baseline metabolic profile, which all vary between patients. This interindividual variability highlights the potential relevance of personalized approaches when considering antioxidant-based strategies in PCOS management [1,12,16].

This review synthesizes current evidence on antioxidants in PCOS, with a focus on OS-related mechanisms, hormonal and metabolic (dys)regulation, and dietary and supplemental antioxidant sources. In addition, the review discusses safety considerations, the role of lifestyle factors, and recent advances in redox biology, while pointing to opportunities for more phenotype-specific and mechanism-oriented antioxidant strategies that could support improved metabolic and reproductive outcomes in PCOS.

## 2. Pathophysiology of PCOS and Oxidative Stress

### 2.1. Mitochondrial Dysfunction and Redox Imbalance

Mitochondria are a major intracellular source of reactive oxygen species (ROS) and are essential for maintaining cellular redox balance. In PCOS, accumulating evidence suggests that mitochondrial dysfunction is associated with increased ROS generation, impaired oxidative phosphorylation, and decreased adenosine triphosphate (ATP) production, processes that may contribute to metabolic inflexibility and the development of insulin resistance [13,17,18]. These disturbances may create a reinforcing cycle in which oxidative stress and insulin resistance amplify one another, further aggravating metabolic dysfunction and endocrine disruption. In this context, mitochondrial impairment may influence not only oxidative damage but also alterations in redox signaling pathways in PCOS [18].

Within the ovarian microenvironment, mitochondrial alterations in granulosa cells appear to be particularly important. Oxidative damage to mitochondrial DNA, altered mitochondrial dynamics, reduced mitochondrial membrane potential, and impaired mitochondrial biogenesis have been reported in granulosa cells from women with PCOS [17,19]. These alterations may compromise granulosa cell function, disrupt follicular development, and reduce oocyte competence. Consistent with these findings, elevated OS levels in follicular fluid have been negatively correlated with embryo quality and reproductive outcomes in women with PCOS [19,20].

Overall, these findings support a mechanistic link between mitochondrial dysfunction, OS, and impaired ovarian function in PCOS, while also highlighting heterogeneity in mitochondrial alterations across individuals.

### 2.2. Hyperandrogenism, Inflammation, and Oxidative Stress

Hyperandrogenism, a defining feature of PCOS, is closely associated with OS, partly through interactions with inflammatory signaling pathways. Experimental and clinical studies indicate that androgen excess may modulate inflammatory signaling pathways, including nuclear factor-κB (NF-κB), increasing production of pro-inflammatory cytokines and enhancing ROS generation [10,21]. This chronic low-grade inflammatory state may further contribute to endothelial dysfunction and systemic metabolic disturbances beyond the ovary [22].

Insulin resistance and compensatory hyperinsulinemia amplify these processes through reciprocal interactions with androgen excess, stimulating ovarian androgen synthesis while promoting oxidative and inflammatory activity in metabolic tissues. Beyond mitochondrial mechanisms discussed above, insulin resistance has also been associated with increased ROS production, mitochondrial dysfunction, and inflammatory signaling, which may worsen endocrine disruption, hyperandrogenism and metabolic dysregulation [13,23,24]. Overall, these interactions establish a dynamic endocrine–metabolic framework in which OS, hyperandrogenism, inflammation, and insulin resistance are closely interrelated and jointly contribute to the maintenance of reproductive and metabolic dysfunction in PCOS [12].

### 2.3. Oxidative Stress, Nitric Oxide Signaling, and Endothelial Dysfunction

Disturbances in nitric oxide (NO) signaling have also been proposed as a mechanism connecting OS with reproductive and vascular abnormalities in PCOS. Increased ROS may lower NO availability by directly reacting with NO and by facilitating the generation of reactive nitrogen species, including peroxynitrite. These processes can impair endothelial function and interfere with normal vascular regulation [10,25,26]. Reduced NO bioavailability may also influence ovarian perfusion and alter conditions within the follicular microenvironment, which could affect follicular maturation and ovulatory processes.

Imbalance between NO and ROS has been associated with ovulatory dysfunction and compromised follicular development in PCOS. Evidence suggests that dysregulated NO signaling, in the context of increased OS, may contribute to impaired folliculogenesis and ovulatory failure, although the contribution of NO-related pathways appears to vary across PCOS phenotypes [10,26].

### 2.4. Redox Signaling Versus Oxidative Damage

At physiological levels, ROS serve essential signaling functions in cellular metabolism, inflammatory responses, and ovarian activity. Under redox homeostasis, ROS-mediated signaling is tightly regulated and necessary for normal follicular development, ovulation, and steroidogenesis [14]. In PCOS, disruption of this balance may shift redox balance toward oxidative damage, including lipid peroxidation and oxidative modification of proteins and nucleic acids, impairing cellular and ovarian function [10,27].

This distinction may help explain heterogeneous findings from antioxidant intervention studies. Non-selective suppression of ROS could potentially disrupt redox-dependent signaling, whereas interventions that support restoration of redox homeostasis may better align with physiological regulatory mechanisms in PCOS [10,12,17].

### 2.5. Evidence of Oxidative Stress in PCOS

Many clinical studies report altered OS profiles in women with PCOS, often characterized by elevated markers of lipid and protein oxidation and reduced antioxidant defense capacity [28,29,30]. Systematic reviews and meta-analyses similarly report increased oxidative damage and impaired antioxidant defenses in PCOS across multiple circulating and tissue-level redox biomarkers [31,32].

It remains unclear whether OS is a primary etiological factor (for example, due to the toxicological burden with elemental endocrine disruptors, such as heavy metals) or a secondary consequence of endocrine and metabolic disturbances in PCOS; however, evidence suggests OS may contribute to disease progression and may represent a potential therapeutic target. Current evidence suggests that OS in PCOS should not be viewed as a single uniform pathological condition but rather as a heterogeneous process that varies among individuals. Multiple factors—including mitochondrial dysfunction, endocrine and inflammatory disturbances, altered NO signaling, and weakened antioxidant defenses—interact across cellular, ovarian, and systemic levels, contributing to substantial variability in redox status among women with PCOS.

This biological complexity may help explain the inconsistent findings reported in antioxidant intervention studies. Approaches that consider metabolic and phenotypic context and focus on restoring redox homeostasis may therefore provide greater insight into treatment responsiveness than strategies aimed at broadly suppressing ROS. These considerations highlight the importance of mechanism-oriented evaluation when assessing antioxidant interventions in PCOS.

## 3. Antioxidants: Classification and Mechanisms of Action in PCOS

### 3.1. Rationale for Antioxidant Classification in PCOS

Considering the mechanistic findings outlined above, organizing antioxidant systems according to functional characteristics may help clarify why responses to interventions in PCOS vary across contexts. Antioxidants represent a heterogeneous group of molecules that differ in chemical structure, redox activity, cellular localization, and biological properties. From a functional perspective, their actions may include direct interaction with reactive species, support of endogenous antioxidant enzyme defenses, or modulation of redox-sensitive signaling pathways. These functional differences arise from variation in molecular targets, intracellular compartmentalization, and the metabolic and inflammatory environment in which these compounds operate [12,33,34,35].

Traditional classification based solely on dietary sources (e.g., vitamins or polyphenols) or supplement use often does not adequately explain the heterogeneous responses reported in PCOS studies. Many compounds commonly described as antioxidants appear to influence cellular processes primarily through regulation of redox-dependent signaling networks rather than through direct scavenging of ROS. The magnitude and direction of these effects depend on several factors, including compound concentration, baseline redox status, cellular distribution, and the integrity of redox-responsive pathways [12,36,37].

Because OS in PCOS differs across phenotypes and metabolic conditions, a classification framework grounded in biological function may provide a more informative basis for interpreting clinical outcomes and mechanistic variability observed in antioxidant research.

### 3.2. Endogenous and Exogenous Antioxidant Systems in PCOS

Maintenance of cellular redox homeostasis depends on coordinated endogenous antioxidant defense systems that control ROS generation, detoxification processes, and redox-related signaling. These systems include enzymatic antioxidants, such as superoxide dismutase, catalase, and glutathione peroxidase, as well as non-enzymatic components, including glutathione and thioredoxin. Their activity is regulated through multiple mechanisms, including transcriptional control, post-translational modification, and broader metabolic regulation [35,38,39]. Redox regulation is further coordinated by stress-responsive transcriptional pathways that control antioxidant gene expression and adaptive cellular responses, linking oxidative stimuli with metabolic and inflammatory context [40].

Alterations in the activity, expression, and regulation of endogenous antioxidant systems have been reported in PCOS, suggesting that impaired redox buffering capacity, in addition to altered ROS generation, may contribute to redox imbalance [10,31]. Reduced antioxidant enzyme activity, altered glutathione metabolism, and disrupted mitochondrial redox balance may contribute to vulnerability to oxidative damage and dysregulated redox signaling [10,31]. The extent of these disturbances differs across PCOS phenotypes and is influenced by factors such as obesity, insulin resistance, and the degree of inflammatory activity.

Exogenous antioxidants obtained through diet or supplementation do not operate independently but interact with endogenous redox systems. Dietary antioxidants may enhance endogenous defense mechanisms, influence redox-sensitive transcriptional pathways, or reduce oxidative damage during periods of increased oxidative load. However, their biological effects depend on several modifying factors, including baseline redox status, overall metabolic health, compound bioavailability, and lifestyle-related influences [39,41,42,43]. In contrast, supplemental antioxidants may produce more specific effects depending on their intracellular distribution and their capacity to influence mitochondrial processes or redox-sensitive signaling pathways, although reported efficacy remains inconsistent across studies [12].

Distinguishing between endogenous and exogenous antioxidant systems may therefore offer a useful mechanistic framework for interpreting why supplementation often produces modest or short-term effects when underlying disturbances in endogenous antioxidant capacity, mitochondrial function, or inflammatory signaling persist [10,31]. This perspective may also help explain why improvements in OS biomarkers do not consistently translate into sustained metabolic or reproductive benefits in clinical investigations [44,45].

### 3.3. Enzymatic and Non-Enzymatic Antioxidants: Functional Implications in PCOS

Endogenous antioxidant defenses are typically categorized into enzymatic and non-enzymatic systems, which differ in both regulatory mechanisms and functional roles in maintaining redox homeostasis. Enzymatic antioxidants operate catalytically to regulate ROS concentrations and prevent oxidative damage. Their activity is tightly controlled through transcriptional regulation, post-translational modification, and metabolic influences [46,47]. Evidence syntheses in PCOS indicate altered expression and activity of several key antioxidant enzymes, changes that are frequently observed alongside insulin resistance and chronic low-grade inflammation [12,21].

Non-enzymatic antioxidants primarily contribute to intracellular redox buffering and modulation of redox-sensitive signaling pathways. Among these, glutathione plays a central role in maintaining cellular redox balance and functions as a substrate for multiple antioxidant enzymes, thereby linking buffering capacity with enzymatic detoxification processes [48]. Disruptions in glutathione-related pathways have been described in PCOS and may contribute to impaired redox regulation and greater vulnerability to oxidative damage at the systemic level, with possible implications for the ovarian microenvironment [21,31].

The distinction between these systems is particularly relevant when interpreting intervention studies. Enzymatic antioxidant activity cannot generally be restored through direct supplementation, as it depends on gene regulation, metabolic conditions, mitochondrial integrity, and the availability of specific micronutrient cofactors [38,39]. In contrast, levels of non-enzymatic antioxidants may be influenced by dietary intake or supplementation. However, their biological effects are often limited by factors such as absorption, cellular transport, and incorporation into endogenous redox networks [41,47]. As a result, interventions aimed at increasing non-enzymatic antioxidants may modify circulating OS biomarkers without necessarily restoring physiologically relevant redox regulation or producing consistent clinical benefit [39,41].

Consequently, evaluation of antioxidant strategies in PCOS requires careful consideration of which components of the antioxidant defense system are being targeted and how these interventions interact with endogenous redox processes. Evidence syntheses examining nutraceutical and micronutrient supplementation report heterogeneous effects across metabolic, endocrine, and OS outcomes, while overall efficacy and safety remain uncertain [49]. Methodological analyses further emphasize that improvements in redox biomarkers do not automatically translate into clinically meaningful benefit, underscoring the importance of linking biochemical indicators with functional outcomes [50]. Translational reviews similarly suggest that redox interventions should be interpreted within the context of integrated antioxidant systems rather than isolated compounds [51]. This functional distinction is therefore central to interpreting evidence on dietary and supplemental antioxidants in PCOS. For clarity, the major endogenous antioxidant systems implicated in PCOS, their primary biological roles, and key regulatory considerations are summarized in Table 1.

### 3.4. Dietary Antioxidants in PCOS

Dietary antioxidants have attracted interest as adjunctive strategies in PCOS because they can be modified through habitual nutrition and may influence oxidative and metabolic homeostasis. However, as they are consumed within complex food matrices and broader dietary patterns, attribution of observed effects to individual compounds remains challenging.

Observational studies and dietary pattern analyses suggest that higher intake of antioxidant-rich foods is associated with more favorable metabolic and inflammatory profiles in women with PCOS, including lower indices of insulin resistance and reduced inflammatory markers [55,56,57,58,59]. However, heterogeneity in dietary assessment methods, outcome measures, and study populations limits consistency across OS biomarkers and reproductive endpoints, and causal relationships remain uncertain [50,60]. These observations highlight the importance of overall dietary quality rather than the effects of individual nutrients in isolation.

Evidence specifically evaluating single antioxidant vitamins in PCOS remains limited. Vitamin E has most frequently been investigated as part of combined supplementation protocols, which in some cases have shown improvements in selected lipid parameters or OS biomarkers, but effects on glycemic control, hormonal status, and anthropometric measures have been inconsistent across studies [61]. In the case of vitamin C, evidence from controlled intervention trials is scarce, and its potential role is more often inferred from dietary intake patterns or circulating antioxidant levels [62]. Carotenoids are generally assessed as components of broader dietary patterns rather than as independent supplements, which makes it difficult to determine their specific contributions [57]. Taken together, these findings suggest that observed associations are unlikely to reflect isolated effects of individual vitamins and should instead be interpreted within the broader context of overall dietary patterns.

Polyphenolic compounds such as curcumin and quercetin have also attracted attention because of their combined antioxidant and anti-inflammatory properties and their potential influence on insulin signaling pathways. Small randomized controlled trials (RCTs) report improvements in selected metabolic or inflammatory markers; however, heterogeneity, limited sample sizes, and short intervention durations preclude firm conclusions regarding clinical efficacy [63,64,65].

The biological activity of dietary antioxidants is further influenced by contextual factors such as food matrix effects, nutrient–nutrient interactions, gut microbiota composition, and baseline redox and metabolic status. These factors modulate intestinal absorption, systemic distribution, metabolism, and downstream redox responses, thereby limiting extrapolation from isolated nutrients or short-term interventions to whole-diet effects [42]. In addition, fermentation of dietary fiber by the gut microbiota generates short-chain fatty acids that can influence inflammatory and redox signaling pathways, further complicating attribution of observed effects to classical antioxidant compounds alone [66]. This complexity is particularly relevant in PCOS, where metabolic heterogeneity and variable oxidative burden are likely to influence responsiveness to dietary interventions.

Importantly, dietary antioxidants rarely act as direct ROS scavengers at physiologically relevant concentrations in vivo [67,68]; instead, their effects are more plausibly mediated through modulation of redox-sensitive signaling pathways and endogenous antioxidant defenses [43,67,69]. These actions are inherently context-dependent and may differ across PCOS phenotypes defined by obesity status, insulin resistance, and degree of hyperandrogenism [10,70].

Overall, dietary antioxidants may modulate OS-related pathways in PCOS, but consistent clinical benefits are not established. Interpretation of dietary studies should account for dietary context, metabolic phenotype, and methodological variability, supporting evaluation of antioxidant intake within integrated dietary patterns rather than isolated nutrients.

### 3.5. Supplemental Antioxidants in PCOS

In contrast to dietary antioxidants, supplemental antioxidants are administered as isolated compounds at defined doses and are often intended to target OS-related pathways or metabolic dysfunction. A variety of supplements has been investigated in women with PCOS, including N-acetylcysteine, coenzyme Q10, α-lipoic acid, melatonin, selenium, and combination formulations [45,49]. These interventions are typically examined for their potential influence on insulin sensitivity, androgen excess, ovulatory function, and circulating markers related to OS and inflammation [10,12,15,71].

Selenium represents a particular case among antioxidant interventions because it is an essential micronutrient with a relatively narrow physiological range. The effects of supplementation are therefore strongly dependent on baseline selenium status, which may partly explain the heterogeneous findings reported across studies [72]. Unlike classical chain-breaking antioxidants, selenium does not primarily act by directly scavenging ROS. Instead, its biological effects arise mainly through incorporation into selenoproteins that support endogenous antioxidant enzyme systems.

Evidence from RCTs and meta-analyses suggests that antioxidant supplementation may lead to modest improvements in certain metabolic and endocrine parameters in PCOS, although results remain inconsistent. For instance, meta-analytic data indicate that N-acetylcysteine may reduce fasting glucose and some lipid parameters [73], while coenzyme Q10 has been associated with favorable effects on lipid metabolism and inflammatory markers in clinical studies [74,75]. In contrast, effects on reproductive outcomes—including ovulation frequency and pregnancy rates—are less consistent, and improvements in OS biomarkers do not reliably correspond to meaningful reproductive benefits [15,76,77].

The variability observed across supplementation trials likely reflects differences in study populations, including baseline metabolic and redox status, obesity severity, and degree of insulin resistance, as well as variation in supplement dosage, duration of intervention, and choice of primary endpoints [78,79,80]. Moreover, many compounds described as antioxidants exert multiple biological actions beyond redox modulation, influencing cellular signaling pathways, energy metabolism, and inflammatory processes. This pleiotropic activity complicates attempts to attribute observed effects solely to antioxidant mechanisms [81]. Importantly, supplementation alone is unlikely to correct underlying disturbances in endogenous antioxidant capacity, mitochondrial function, or chronic inflammatory signaling that contribute to PCOS pathophysiology [44,80].

Considerations related to safety further restrict the broader interpretation of supplementation studies. Evidence regarding the long-term effects of high-dose or prolonged antioxidant use in women with PCOS remains limited, and excessive antioxidant exposure may interfere with physiological redox signaling or adaptive metabolic responses [51,82]. Potential interactions with endocrine and metabolic pathways, together with marked interindividual variability in responsiveness, underscore the need for caution when extrapolating short-term trial results to routine clinical use.

Overall, supplemental antioxidants may confer metabolic or biochemical benefits in selected subgroups of women with PCOS, particularly those with pronounced insulin resistance or elevated oxidative burden; however, available evidence does not support uniform or indiscriminate use. Clinical responsiveness appears dependent on metabolic context, phenotype, and intervention duration. Accordingly, evaluation of antioxidant strategies in PCOS should prioritize clinically relevant metabolic, hormonal, and reproductive outcomes rather than changes in OS biomarkers alone. Key antioxidant supplements investigated in PCOS, along with proposed mechanisms and reported limitations, are summarized in Table 2.

Collectively, these findings indicate that antioxidant supplementation in PCOS primarily influences downstream redox and metabolic markers, with clinical responsiveness strongly dependent on metabolic context, phenotype, and intervention duration. Accordingly, evaluation of antioxidant strategies in PCOS requires careful consideration of clinical trial evidence, including metabolic, hormonal, and reproductive outcomes, rather than reliance on changes in OS biomarkers alone.

## 4. Clinical Evidence of Antioxidant Interventions in PCOS

### 4.1. Overview of Clinical Evidence

Clinical evidence evaluating antioxidant interventions in women with PCOS is derived primarily from RCTs and meta-analyses assessing short-term metabolic, endocrine, inflammatory, and OS-related outcomes [44,70,80]. Across compounds, intervention durations are typically limited to several weeks or months, with modest sample sizes and substantial variability in participant characteristics, including obesity status, baseline insulin resistance, and concomitant therapies.

Outcome selection differs considerably between studies. Many RCTs prioritize metabolic indices or biochemical OS markers, whereas endocrine outcomes are assessed less consistently and reproductive endpoints are frequently secondary or insufficiently powered [44,76,84]. Consequently, the clinical relevance and long-term applicability of available findings remain uneven.

Heterogeneity in study design, supplement formulation and dosing, comparator selection, and baseline nutritional status further limits comparability across RCTs and complicates pooled effect estimation [44,76,84]. These methodological limitations necessitate cautious interpretation and support outcome-specific evaluation rather than reliance on isolated biomarkers or individual trial findings.

### 4.2. Metabolic Outcomes

#### 4.2.1. Insulin Sensitivity and Glycemic Control

Insulin resistance-related outcomes are frequently reported as primary or secondary endpoints in antioxidant trials in PCOS; however, the evidence base remains inconsistent and often of limited certainty. Meta-analyses indicate that some interventions are associated with statistically significant changes in surrogate indices of insulin sensitivity, such as homeostasis model assessment of insulin resistance (HOMA-IR) or quantitative insulin sensitivity check index (QUICKI), but findings are highly heterogeneous, sometimes driven by a small number of trials, and frequently graded as low or very low certainty [44,70,80,84].

Across analyses, statistically significant effects are often confined to isolated surrogate indices or fasting markers, with limited concordance among fasting glucose, fasting insulin, and derived indices. For example, α-lipoic acid reduced HOMA-IR without a significant pooled change in fasting insulin [84], whereas N-acetylcysteine reduced fasting glucose without improving fasting insulin or HOMA-IR [73]. Melatonin improved total antioxidant capacity but showed no pooled effect on glycemic parameters [76]. The lack of consistency across related endpoints limits interpretation, as changes restricted to individual markers may reflect analytical variability or between-trial differences rather than clinically meaningful improvement in insulin action.

Metabolic responses also appear influenced by baseline phenotype. Subgroup analyses suggest greater improvements in insulin sensitivity indices among overweight women with PCOS than among obese women, indicating reduced responsiveness in more advanced metabolic dysfunction [89]. These differences likely reflect variation in the percentage of adipose tissue in the body mass composition, with consequent variation in underlying metabolic severity and redox status across phenotypes. It is known that adipose tissue plays a central role in the sequestration (storage) of fat-soluble supplements like vitamin D, E, and A, reducing their circulating bioavailability. This causes their faster clearance from the blood and reliance on adipose release, which is inefficient. Furthermore, in overweight women, metabolic disturbances may be less advanced and more responsive to interventions targeting insulin signaling and oxidative pathways, whereas in obese individuals, greater insulin resistance, chronic inflammation, and mitochondrial dysfunction may limit responsiveness to antioxidant supplementation alone. Adiposity-related factors, including also altered adipokine signaling and increased systemic oxidative burden, may further contribute to reduced therapeutic effects in more metabolically compromised phenotypes. However, these analyses are limited by small sample sizes and inconsistent methodology. Several meta-analyses further report substantial heterogeneity, with pooled estimates sensitive to trial selection and risk-of-bias exclusions. These observations support the need for phenotype-informed and individualized approaches when evaluating antioxidant strategies in PCOS, rather than applying uniform supplementation protocols across heterogeneous patient populations.

Overall, current evidence does not support a uniform or reliable effect of antioxidant supplementation on insulin resistance in PCOS. Improvements in surrogate indices should therefore be interpreted cautiously, given short intervention durations, absence of long-term follow-up, and inconsistent effects on clinically relevant glycemic outcomes.

#### 4.2.2. Lipid Profile

Compared with insulin resistance-related outcomes, the effects of antioxidant interventions on lipid parameters in women with PCOS are less consistent and generally modest. Systematic reviews and meta-analyses of RCTs report heterogeneous and frequently null pooled effects on total cholesterol (TC), low-density lipoprotein cholesterol (LDL-C), high-density lipoprotein cholesterol (HDL-C), triglycerides (TG), and very-low-density lipoprotein cholesterol (VLDL) across antioxidant classes and study designs [70,76].

When statistically significant changes are reported, they are typically restricted to individual lipid fractions, specific supplements, or selected subgroups and are not consistently reproduced across analyses [70,76,80,84]. In several syntheses, improvements in OS biomarkers occur without corresponding changes in lipid parameters, suggesting that short-term redox modulation alone is insufficient to induce clinically meaningful alterations in lipid metabolism over typical intervention durations [76,85].

Interpretation is further limited by methodological factors. Many RCTs include participants without baseline dyslipidemia, are underpowered for lipid outcomes, or are too short to capture metabolic adaptation. Furthermore, inadequate control of dietary intake, body weight variation, and concomitant treatments in many trials complicates the interpretation of whether observed outcomes can be directly attributed to antioxidant supplementation.

Taken together, current evidence does not support a consistent lipid-modifying effect of antioxidant supplementation in PCOS. Reported improvements are small, heterogeneous, and population-dependent, indicating that lipid management in PCOS is unlikely to be meaningfully influenced by antioxidant supplementation in the absence of broader metabolic and lifestyle interventions.

### 4.3. Hormonal and Endocrine Outcomes

Endocrine outcomes in antioxidant intervention studies of PCOS generally show weaker and less reproducible responses than metabolic endpoints. Across systematic reviews and meta-analyses of RCTs, pooled effects on circulating androgens, gonadotropins, and sex hormone-binding globulin (SHBG) are frequently null, modest, or highly heterogeneous. When statistically significant effects are observed, they are typically confined to isolated hormonal markers, specific subgroups, or particular study contexts and are not consistently reproduced across analyses [80].

Evidence from individual antioxidant classes reflects this broader pattern. Meta-analysis of melatonin supplementation demonstrated no significant pooled effects on total testosterone, SHBG, or clinical hyperandrogenism indices despite increases in total antioxidant capacity (TAC), indicating dissociation between redox biomarker changes and endocrine responses [76]. Meta-analytic evidence for α-lipoic acid supplementation likewise indicates no significant overall effects on testosterone, luteinizing hormone (LH), follicle-stimulating hormone (FSH), or estrogen, and substantial heterogeneity between studies has been reported [84]. Similarly, broader evidence syntheses that evaluate antioxidants collectively describe only minimal effects on testosterone despite modest improvements in some metabolic parameters [80].

Evidence regarding selenium supplementation is also inconsistent. While certain analyses report improvements in oxidative or inflammatory biomarkers, pooled endocrine outcomes are frequently heterogeneous or non-significant, which may reflect differences in baseline selenium status and the limited number of available trials [90].

Individual RCTs have occasionally described hormonal changes in specific contexts. For example, reductions in testosterone have been observed with co-supplementation of coenzyme Q10 and vitamin E, suggesting that endocrine effects may depend on formulation and study conditions rather than representing a consistent biological response [91].

Interpretation of these findings is further complicated by several methodological factors, including short intervention durations, heterogeneity among PCOS phenotypes, frequent assessment of hormonal variables as secondary outcomes, and variability in laboratory assays and sampling protocols. Taken together, the current evidence does not support antioxidant supplementation as a reliable approach for correcting the core endocrine disturbances associated with PCOS. Hormonal responses appear to be strongly context-dependent and should therefore be interpreted alongside metabolic status, phenotype, comparator therapy, and intervention duration rather than viewed as primary indicators of therapeutic efficacy.

### 4.4. Reproductive Outcomes

Reproductive endpoints are among the most clinically meaningful outcomes in studies of PCOS, yet they remain the least consistently evaluated in trials investigating antioxidant interventions. Evidence from RCTs and meta-analyses assessing ovulation, pregnancy, and live birth is generally limited and highly heterogeneous. In many studies, these outcomes are secondary measures, often assessed in relatively small samples and frequently in combination with fertility treatments, which makes it difficult to isolate the specific contribution of antioxidant supplementation [76,80,84].

The strength of available evidence also varies across antioxidant compounds and depends on the comparator used. For example, a meta-analytic evaluation of N-acetylcysteine has shown higher odds of ovulation and pregnancy compared with placebo, but lower odds when compared with metformin. This comparator-dependent pattern limits its interpretation as a primary intervention for improving fertility outcomes [89]. For melatonin, pooled analyses found no significant effect on pregnancy rate despite improvements in OS biomarkers and occasional increases in endometrial thickness, while live birth outcomes were largely unavailable [76]. Similarly, meta-analyses of α-lipoic acid report insufficient evidence for ovulation or pregnancy outcomes because most RCTs prioritized metabolic endpoints [84].

Data for other antioxidants remain sparse. Studies of coenzyme Q10 suggest possible improvements in clinical pregnancy rates in selected assisted reproduction contexts; however, live birth outcomes are inconsistently reported and overall certainty of evidence is low [83,92]. For selenium, reproductive outcomes are rarely assessed, and current evidence is insufficient to determine fertility effects in women with PCOS [85].

Interpretation is further constrained by frequent co-interventions, including ovulation induction therapies, assisted reproductive technologies, and combination nutraceutical regimens, as well as short intervention durations and limited follow-up to live birth. Overall, available evidence does not support antioxidant supplementation as a reliable standalone strategy for improving fertility outcomes in PCOS. Reported reproductive effects are inconsistent, comparator-dependent, and generally weaker than metabolic responses, indicating that any potential benefit is likely indirect and mediated through broader metabolic or therapeutic context rather than direct restoration of reproductive function. Future studies should prioritize clinically meaningful reproductive endpoints, particularly live birth rates, as primary outcomes rather than secondary measures, to better define the clinical relevance of antioxidant interventions in PCOS.

### 4.5. Oxidative Stress Biomarkers vs. Clinical Endpoints

Changes in OS biomarkers are among the most frequently reported outcomes in antioxidant intervention studies of PCOS. Findings from RCTs and meta-analyses indicate that antioxidant supplementation is frequently associated with statistically significant changes in circulating redox markers, including malondialdehyde, total antioxidant capacity (TAC), and reduced glutathione [76,84]. Nevertheless, these biochemical shifts are not consistently accompanied by improvements in metabolic, endocrine, or reproductive outcomes.

Evidence syntheses repeatedly highlight a disconnect between changes in redox biomarkers and clinically meaningful effects. Meta-analyses examining interventions such as melatonin, α-lipoic acid, selenium, and N-acetylcysteine report that improvements in OS indicators often occur without corresponding reductions in insulin resistance, androgen levels, ovulatory dysfunction, or pregnancy outcomes [76,84,85,92]. These findings suggest that short-term alterations in systemic oxidative status alone may be insufficient to influence the complex endocrine–metabolic disturbances characteristic of PCOS.

Interpretation of these results is further constrained by the use of OS markers as surrogate endpoints. Many circulating biomarkers reflect overall redox balance rather than oxidative processes occurring within specific metabolically relevant tissues, such as adipose tissue, skeletal muscle, or the ovarian microenvironment [21,93]. In addition, differences across studies in biomarker selection, analytical techniques, and timing of assessment limit comparability and complicate clinical interpretation of pooled findings [93,94].

These observations are consistent with current concepts in redox biology, which emphasize restoration of redox homeostasis rather than indiscriminate suppression of ROS [33,95]. In this context, the therapeutic relevance of antioxidant interventions may depend less on direct ROS scavenging and more on their capacity to modulate endogenous antioxidant defense systems, including enzymatic pathways such as superoxide dismutase, catalase, and glutathione-related mechanisms, which are integral to maintaining redox homeostasis in PCOS. Accordingly, changes in circulating OS markers should be considered supportive mechanistic indicators rather than reliable predictors of clinical response, particularly when upstream drivers such as insulin resistance, adiposity, and chronic inflammation remain unmodified [7,96].

Overall, antioxidant supplementation consistently alters biochemical redox parameters but produces modest and heterogeneous clinical effects. Future RCTs should therefore prioritize clinically meaningful outcomes and interpret biomarker changes within integrated metabolic and phenotypic contexts rather than as standalone efficacy measures, highlighting the limitations of relying on OS biomarkers as surrogate endpoints and the need to link biochemical changes with long-term clinical outcomes. This apparent disconnect may be explained by the limited tissue specificity of circulating biomarkers, the persistence of upstream metabolic and inflammatory drivers, and the possibility that short-term modulation of systemic redox status is insufficient to induce clinically meaningful endocrine or reproductive changes.

### 4.6. Safety, Duration, and Clinical Applicability

Across antioxidant intervention studies in PCOS, safety and tolerability are generally acceptable over the short durations typical of available RCTs. Most interventions are evaluated over periods ranging from several weeks to a few months, and serious adverse events are uncommon. However, reporting of adverse effects is frequently incomplete, and the absence of reported harm should not be interpreted as evidence of long-term safety [76,92].

A major limitation of the evidence base is the short duration of most RCTs. Given the chronic nature of PCOS and its metabolic and reproductive consequences, short-term improvements in biochemical or surrogate outcomes may not translate into sustained clinical benefit or long-term safety. Few studies include post-intervention follow-up, and data on durable metabolic, endocrine, or reproductive outcomes remain limited [85].

Safety considerations also differ by compound. For supplements such as N-acetylcysteine and coenzyme Q10, available evidence suggests an acceptable short-term safety profile in women with PCOS; however, optimal dosing, long-term use, and potential interactions with endocrine or metabolic therapies remain insufficiently characterized [89,97,98]. Selenium represents a particular case among antioxidant interventions because it has a relatively narrow therapeutic window. The effects of supplementation are highly dependent on baseline selenium status, and both deficiency and excessive intake may lead to adverse outcomes, which complicates the interpretation of study findings and limits their generalizability [85].

The translation of research findings into clinical practice is also restricted by substantial heterogeneity across studies. Differences in participant characteristics, intervention protocols, and outcome measures make comparisons difficult. In addition, many RCTs include selected study populations, exclude individuals with certain comorbidities, or allow concurrent lifestyle and pharmacological interventions, which makes it challenging to isolate the effects attributable specifically to antioxidant supplementation. Interindividual variation in metabolic phenotype, nutritional status, and baseline oxidative burden may further influence both efficacy and safety, suggesting that uniform supplementation strategies may not be appropriate for all women with PCOS [7].

Taken together, the available evidence supports a cautious and individualized approach when considering antioxidant supplementation in PCOS. Although short-term supplementation appears generally well tolerated, lack of long-term safety data, uncertain durability of clinical outcomes, and substantial patient variability preclude broad, uniform recommendations. When considered, antioxidant therapy should be integrated into comprehensive lifestyle and metabolic management rather than used as a standalone intervention, and guided by phenotype, baseline nutritional status, and clearly defined clinical objectives. In particular, the long-term safety of high-dose antioxidant supplementation remains poorly defined, and further research is required to evaluate potential adverse effects, optimal dosing, and interactions with physiological redox signaling. Table 3 summarizes response patterns and key limitations across major outcome domains discussed in this section.

### 4.7. Clinical Considerations and Practical Recommendations for Antioxidant Use in PCOS

From a clinical perspective, antioxidant interventions in PCOS should be considered as adjuncts to first-line strategies rather than stand-alone therapies [1,80].

Despite heterogeneity across studies, some findings may still offer clinically relevant guidance in selected patient subgroups [44,80].

In women with predominant insulin resistance or metabolic dysfunction, N-acetylcysteine (approximately 1200–1800 mg/day) and α-lipoic acid (600–1800 mg/day) have shown evidence of benefit for selected surrogate markers of glycemic control, although findings remain heterogeneous across studies [73,84]. Coenzyme Q10 (100–300 mg/day) may provide additional metabolic benefit in some women with PCOS, although evidence remains limited and phenotype-specific effects are not yet well defined [74,75].

Melatonin (3–10 mg/day) has been investigated mainly in relation to circadian regulation and the ovarian microenvironment, although evidence for meaningful reproductive outcomes remains limited [76,77]. Selenium supplementation (~200 µg/day) may influence oxidative and inflammatory markers; however, its use requires caution due to its narrow therapeutic range and dependence on baseline selenium status [85,86,87,88,90].

Routine or indiscriminate use of antioxidant supplementation cannot be recommended. Clinical decisions should be individualized, taking into account metabolic profile, dietary context, and potential deficiencies [44,80].

## 5. Gut Microbiota, Oxidative Stress, and Antioxidant Responsiveness in PCOS

The heterogeneity and context-dependence observed in antioxidant intervention studies (Section 4) suggest that upstream biological modifiers may influence OS burden and therapeutic responsiveness in PCOS. Emerging, but still largely associative, evidence indicates that the gut microbiota may operate at the intersection of metabolism, inflammation, endocrine regulation, and redox homeostasis in PCOS; however, causal relationships and mechanistic directionality remain incompletely defined. Consequently, microbiota-related mechanisms are increasingly considered potential modulators of oxidative and metabolic pathways, although current understanding is derived primarily from indirect and cross-sectional observations.

### 5.1. Gut Microbiota Alterations and Oxidative Stress in PCOS

Building on evidence linking OS to metabolic and endocrine dysfunction in PCOS, recent research has identified the gut microbiota as a potential upstream modulator of redox homeostasis. The intestinal microbiota contributes to host metabolic and endocrine regulation not only through nutrient metabolism and immune signaling but also via its influence on redox balance and antioxidant defense pathways [99,100,101].

On one hand, for example, enrichment of lipopolysaccharide (LPS) producing *Proteobacteria* in PCOS, specifically increased relative abundance of *Enterobacteriaceae*, has been associated with metabolic endotoxemia, TLR4 activation, and ROS generation via NADPH oxidases, along with potential suppression of Nrf2-dependent antioxidant pathways [102,103]. Alterations in microbial composition and functional capacity may therefore aggravate PCOS pathophysiology by increasing oxidative burden and impairing endogenous antioxidant systems.

On the other hand, dietary antioxidants may beneficially influence gut microbiota composition and function (metabolite production) through several mechanisms, including changes in microbial diversity, selective enrichment of beneficial taxa, enhancement of antioxidant enzyme activity, and modulation of microbial metabolite production [104,105]. Polyphenols and related bioactive compounds can be metabolized by intestinal microbes, resulting in the generation of metabolites such as short-chain fatty acids, which may contribute to redox regulation and inflammatory signaling [104,105]. In addition, antioxidant compounds may affect intestinal barrier function and local OS within the gut environment, thereby indirectly shaping microbial composition [106]. However, these interactions are context-dependent and remain insufficiently characterized in PCOS-specific populations [105].

However, current evidence is predominantly associative and largely derived from cross-sectional or short-term intervention studies, which limits the ability to establish causal relationships between microbiota alterations, OS, and clinical outcomes in PCOS.

### 5.2. Metabolic and Inflammatory Pathways Linking Dysbiosis and Oxidative Stress

Gut microbiota dysbiosis has been associated with impaired glucose and lipid handling, insulin resistance, endocrine disruption and altered hormone secretion—processes central to PCOS pathophysiology that may be influenced by microbial activity [101,107,108]. Within this framework, OS represents a mechanistic interface through which microbiota-related disturbances may interact with established metabolic abnormalities rather than an independent initiating factor.

Women with PCOS often exhibit lower α-diversity (species richness and evenness), especially in hyperandrogenic and obese subgroups. Low diversity further correlates with higher systemic inflammation (C-reactive protein, interleukin 18) and OS markers, as seen in metabolic-inflammatory PCOS phenotypes. Many PCOS cohorts show increased *Bacteroidetes*/*Firmicutes* ratio, or other imbalances in these phyla, which are associated with insulin resistance, lipid dysregulation, and chronic inflammation, all amplifying oxidative burden [109].

Some PCOS groups show increased *Bifidobacterium*, often in hyperandrogenic PCOS. In certain contexts, elevated *Bifidobacterium* correlates with abnormal steroid metabolism and altered bile acid profiles, which may indirectly worsen insulin resistance and OS, although the exact mechanism is still being clarified [102].

Higher *Streptococcus*, *Enterococcus*, and *Eubacterium nodatum* groups have been reported in some studies to be enriched in hyperandrogenic PCOS. These taxa are often associated with mucosal inflammation, barrier disruption, and pro-inflammatory metabolites, which can amplify ROS and decrease antioxidant enzyme activity (superoxide dismutase, catalase) in tissues [110].

PCOS dysbiotic profiles frequently include increased abundance of H_2_S-producing bacteria (e.g., *Desulfovibrio*-like). Elevated sulphur-reducing bacteria overproduce H_2_S, and excess H_2_S inhibits mitochondrial complex IV, increases lactate and ROS, and may deplete glutathione, contributing to ovarian and systemic OS [111].

Although *Prevotella* can be pro-inflammatory in other conditions, its reduced abundance in PCOS cohorts, compared to controls, is often associated with loss of fiber-fermenting capacity and short-chain fatty acids-mediated antioxidant support, indirectly favoring OS [111].

Lower abundance of protective, antioxidant-supporting taxa, like *Faecalibacterium*, *Agathobacter*, *Ruminococcus* and other *Ruminococcaceae*/*Lachnospiraceae* butyrate producers, especially in high-testosterone PCOS, via loss of butyrate, reduces activation of Nrf2, SIRT1, and regulatory T cells, weakening superoxide dismutase, catalase, and glutathione defences and increasing epithelial ROS. This depletion links PCOS-related dysbiosis to OS and metabolic dysfunction, which are spread along a gut–liver–ovary axis [112].

Insulin resistance, present in approximately 50–70% of women with PCOS, provides a metabolic substrate upon which redox and inflammatory signaling may converge [113]. Altered microbial composition may contribute to oxidative burden by reducing the availability of microbial-derived antioxidant metabolites while promoting pro-inflammatory signaling, thereby modulating insulin signaling efficiency and downstream androgen metabolism [101,107].

These metabolic alterations are closely associated with chronic low-grade inflammation that may arise from compromised intestinal barrier function. Reduced *Akkermansia* in PCOS, especially in obese women, weakens the mucin barrier, promoting lipopolysaccharide leakage and systemic inflammation, which further increases oxidative burden and impairs antioxidant capacity [114]. Increased intestinal permeability can permit the translocation of inflammatory molecules into the systemic circulation, thereby amplifying oxidative and inflammatory signaling in metabolically vulnerable individuals [100,106]. Current evidence indicates that metabolic disturbances, inflammation, and OS interact in a bidirectional and context-dependent manner rather than following a simple causal pathway driven exclusively by microbial alterations.

Importantly, longitudinal and interventional studies specifically designed to assess causal pathways linking antioxidant exposure, microbiota modulation, and clinical outcomes in PCOS are currently limited. This represents a key gap in the literature and restricts the translation of microbiota-related findings into targeted therapeutic strategies.

### 5.3. Neuroendocrine Considerations and the Gut–Brain Axis

In addition to metabolic and inflammatory mechanisms, the gut microbiota has been suggested to influence PCOS-related features through interactions with neuroendocrine regulation. Communication along the gut–brain axis allows microbial signals to affect hypothalamic–pituitary activity, stress responses, appetite control, and energy balance—processes that are relevant to PCOS pathophysiology.

OS has been linked to dysregulation of hypothalamic–pituitary function in broader neuroendocrine settings; however, its specific role in mediating gut–brain interactions in PCOS is not yet well defined. Changes in redox balance associated with alterations in the gut microbiota may influence neuroendocrine signaling and indirectly contribute to hormonal and metabolic disturbances characteristic of PCOS, although direct evidence from human studies remains limited [100].

For this reason, gut–brain–redox interactions in PCOS should currently be considered biologically plausible but largely inferential, with limited direct human evidence and insufficient longitudinal data to confirm mechanistic or causal relationships.

## 6. Lifestyle Integration, Antioxidants, and Environmental Considerations—Future Perspectives

Lifestyle modification, including dietary improvement and regular physical activity, continues to represent the primary strategy in PCOS management because of its well-documented benefits for metabolic, reproductive, and psychological health. Evidence increasingly suggests that antioxidant strategies may act as supportive measures when combined with lifestyle interventions rather than as independent therapies. Some studies report additional improvements in body composition, insulin resistance, and androgen-related parameters when antioxidant approaches accompany lifestyle modification; however, substantial variability in intervention design, duration, and adherence makes it difficult to determine the magnitude and persistence of these potential synergistic effects [78].

Dietary patterns emphasizing low glycaemic index foods, higher dietary fiber intake, and Mediterranean-style eating habits have been associated with favorable metabolic and endocrine outcomes in women with PCOS [1]. The potential benefits of these dietary patterns are unlikely to arise solely from direct antioxidant intake. Instead, they may influence endogenous redox regulation, reduce inflammatory burden, and interact with gut microbiota-derived metabolic pathways. Similarly, regular physical activity—including both aerobic exercise and resistance training—has been shown to improve insulin sensitivity, decrease central adiposity, and support cardiovascular and reproductive health in women with PCOS [1,115]. Nevertheless, responses to lifestyle interventions vary considerably among individuals, underscoring the importance of phenotype-informed and personalized treatment strategies [116].

Environmental exposures may represent an additional factor influencing oxidative burden in PCOS. Growing evidence links endocrine-disrupting chemicals, such as bisphenols, phthalates, organochlorines, and certain heavy metals, with disturbances in steroidogenesis, insulin signaling, and ovarian function [117,118,119]. These compounds have also been associated with increased ROS generation, mitochondrial dysfunction, and impairment of endogenous antioxidant defense mechanisms. In this context, OS may act as a mechanistic interface connecting environmental exposures with PCOS-related metabolic and reproductive disturbances [120,121,122,123]. Although available human evidence is largely observational and heterogeneous, considering environmental influences may help explain differences in oxidative status and variability in therapeutic response.

Future investigations should move beyond reductionist models that treat antioxidants solely as ROS-scavenging agents and instead adopt integrated redox–metabolic frameworks that reflect the biological complexity of PCOS. Longitudinal research combining microbiome profiling, metabolomic analyses, environmental exposure assessment, and tissue-relevant OS biomarkers may help clarify causal relationships and identify patient subgroups most likely to benefit from targeted interventions [93,95]. At the same time, safety considerations remain essential. The long-term effects of high-dose or prolonged supplementation strategies—including certain micronutrients and emerging nicotinamide adenine dinucleotide (NAD^+^)-modulating compounds—require further evaluation with respect to appropriate dosing, bioavailability, and clinical outcomes [124,125].

Overall, incorporating antioxidant strategies into comprehensive lifestyle-based management, while focusing on restoration of redox homeostasis rather than indiscriminate suppression of ROS, represents a promising direction for future PCOS research and clinical practice.

At the same time, the substantial heterogeneity observed across studies highlights the need for greater standardization of outcome selection in antioxidant research in PCOS. Development of a core outcome set, including key metabolic (e.g., insulin sensitivity), endocrine (e.g., androgen levels), reproductive (e.g., ovulation and live birth rates), and clinically relevant patient-centered outcomes, would facilitate comparison across studies and improve the quality of evidence synthesis. Standardization of intervention protocols, including dosage, duration, and reporting of baseline nutritional and metabolic status, would further enhance interpretability and support more robust meta-analytic evaluation of antioxidant strategies in PCOS.

## 7. Conclusions

PCOS is a multifaceted endocrine–metabolic disorder in which OS constitutes one element of a broader pathophysiological network involving insulin resistance, chronic low-grade inflammation, mitochondrial dysfunction, endocrine disruption, and disturbances in ovarian signaling. Evidence increasingly suggests that alterations in redox homeostasis arise from complex interactions between metabolic and reproductive pathways rather than from a single primary pathogenic factor.

Antioxidant interventions frequently produce detectable changes in circulating OS biomarkers and, in some cases, modest improvements in metabolic indicators. However, consistent benefits for endocrine abnormalities or reproductive outcomes have not been demonstrated across studies. Variability in findings indicates that treatment responsiveness is influenced by multiple factors, including baseline metabolic status, degree of adiposity, dietary patterns, and individual differences in redox balance. Consequently, antioxidant strategies are likely to have greater relevance when incorporated as supportive components of comprehensive lifestyle-based management rather than used as isolated therapeutic interventions.

Future investigations should prioritize phenotype-specific, metabolically stratified, and mechanistically informed approaches in order to better account for heterogeneity in treatment response and enhance clinical applicability. Longitudinal studies integrating microbiome assessment and clinically meaningful endpoints beyond surrogate redox biomarkers will be essential for clarifying the therapeutic potential of antioxidant strategies in PCOS. In particular, approaches aimed at restoring physiological redox regulation, rather than broadly suppressing ROS, may improve interpretation of clinical trial results and contribute to more individualized management of PCOS. These strategies may be particularly relevant when focused on the modulation of endogenous antioxidant defense systems, including enzymatic pathways such as superoxide dismutase, catalase, and glutathione-related mechanisms. Greater emphasis should also be placed on long-term, well-designed studies evaluating clinically relevant end-points, including live birth outcomes, alongside comprehensive safety assessment. In parallel, the development of phenotype-specific antioxidant strategies and standardized dosing protocols based on clinically meaningful metabolic and reproductive outcomes will be essential.

## Figures and Tables

**Table 1:** Major endogenous antioxidant systems implicated in PCOS: functional characteristics and regulatory considerations.

Antioxidant System	Representative Components	Primary Biological Role	Cellular Localization	Relevance to PCOS	Key Regulatory Considerations
Enzymatic antioxidant systems [31,52]	superoxide dismutase, catalase, glutathione peroxidase	catalytic regulation of ROS levels; limitation of oxidative damage; maintenance of redox balance	cytosol, mitochondria, peroxisomes	reduced activity and altered regulation reported in PCOS; often associated with insulin resistance and chronic low-grade inflammation	activity depends on gene expression, post-translational regulation, mitochondrial integrity, and availability of micronutrient cofactors (e.g., Se, Cu, Zn)
Glutathione-dependent redox system [48,53]	reduced glutathione, glutathione reductase, glutathione transferases	intracellular redox buffering; detoxification of peroxides; maintenance of redox signaling	cytosol, mitochondria	altered glutathione-related antioxidant capacity reported in PCOS at systemic and ovarian levels; increased susceptibility to oxidative damage	regulated by biosynthesis, recycling (glutathione/glutathione disulfide), NADPH availability, and nutritional status
Thioredoxin system [54]	thioredoxin, thioredoxin reductase, peroxiredoxins	modulation of redox-sensitive signaling pathways; protection against oxidative stress	cytosol, mitochondria, nucleus	implicated in redox signaling dysregulation and inflammatory processes relevant to PCOS	sensitive to oxidative load and metabolic context

Abbreviations: PCOS—polycystic ovary syndrome; ROS—reactive oxygen species; Se—selenium; Cu—copper; Zn—zinc; NADPH—nicotinamide adenine dinucleotide phosphate.

**Table 2:** Key antioxidant supplements studied in PCOS: mechanistic targets, evidence signals, and limitations affecting clinical generalizability.

Supplement	Typical Trial Dose Range and Duration	Dominant Mechanistic Targets Relevant to PCOS	Outcomes with Most Reproducible Signal	Outcomes Inconsistent or Insufficient	Generalizability Constraints	Safety and Monitoring Considerations
N-acetylcysteine [16,73]	varied; ~5 days to 24 weeks	glutathione precursor and thiol redox modulator; anti-inflammatory and cytoprotective actions; influences insulin signaling, mitochondrial function, and redox-sensitive pathways beyond direct ROS scavenging	insulin sensitivity indices and glycemic markers (variable across trials)	BMI, androgen levels, menstrual regularity, and live-birth outcomes	marked heterogeneity in dose, duration, phenotype, baseline insulin resistance, and comparator therapy; frequent combination regimens limit attribution to N-acetylcysteine alone; short follow-up predominates	generally well tolerated; optimal dosing unclear; potential for excessive antioxidant exposure to affect physiological redox signaling with high-dose or prolonged use
Coenzyme Q10 [83]	varied; typically 8–24 weeks	lipid-soluble quinone involved in mitochondrial electron transport and bioenergetics; membrane-associated antioxidant activity; may influence insulin signaling, lipid metabolism, and endocrine pathways	insulin resistance indices and selected lipid parameters (dependent on formulation and co—interventions)	reproductive endpoints (ovulation, pregnancy), long-term outcomes, and some endocrine parameters	heterogeneity in dose and formulation (alone vs combinations), phenotype, trial duration, and endpoints; many trials short-term	generally well tolerated; long-term safety in PCOS not well defined; formulation and bioavailability differences may influence effects
α-lipoic acid [84]	600–1800 mg/day; 6–25 weeks	mitochondrial and cytosolic redox modulator; improves glucose transport/insulin signaling (e.g., GLUT-4 translocation, AMPK activation); modulates redox-sensitive metabolic pathways rather than acting solely as a ROS scavenger	fasting blood glucose (moderate certainty) and HOMA-IR (low certainty) reductions, particularly in insulin-resistant women	BMI, fasting insulin, lipid profile, sex hormones (FSH, LH, testosterone, estrogen), and global OS biomarkers (MDA, TAC)	between-study heterogeneity; small samples; short durations; variable dosing; limited reproductive outcome data; frequent combination with inositol complicates attribution	generally well tolerated short-term; long-term safety and optimal dosing in PCOS uncertain
melatonin [76]	3–10 mg/day; 3–12 weeks	endogenous circadian and ovarian regulatory hormone exhibiting direct and indirect antioxidant activity; modulates redox-sensitive inflammatory pathways and follicular microenvironment	increase in TAC	glycemic markers (fasting glucose, insulin, HOMA-IR), lipid profile, inflammatory biomarkers (hs-CRP, MDA), androgen levels, clinical hyperandrogenism indices, and reproductive outcomes remain inconsistent and frequently underpowered	small number of RCTs; short durations; moderate heterogeneity; limited geographic representation; reproductive endpoints often secondary	generally well tolerated in short-term studies; long-term safety and clinical efficacy beyond modulation of OS remain uncertain
selenium [71,85,86,87,88]	commonly ~200 µg/day; typically 8–12+ weeks	essential trace element incorporated into selenoproteins (e.g., glutathione peroxidases); supports antioxidant enzyme function and modulates inflammatory pathways	signals reported for reductions in selected OS and inflammatory biomarkers (e.g., MDA, hs-CRP) and modest improvements in insulin sensitivity indices in some analyses	effects on fasting glucose, insulin, HOMA-IR, androgen levels, lipid profile, TAC, glutathione, NO, SHBG, and clinical hyperandrogenism scores inconsistent across meta-analyses and individual RCTs	small and heterogeneous RCTs; variable doses and durations; short follow-up; limited reproductive outcome data; differences across meta-analyses in biomarker selection and analytical approach	generally well tolerated at studied doses; narrow therapeutic index; long-term safety and optimal dosing in women with PCOS remain poorly defined

Abbreviations: PCOS—polycystic ovary syndrome; ROS—reactive oxygen species; BMI—body mass index; HOMA-IR—homeostasis model assessment of insulin resistance; GLUT-4—glucose transporter type 4; AMPK—adenosine monophosphate-activated protein kinase; FSH—follicle-stimulating hormone; LH—luteinizing hormone; MDA—malondialdehyde; TAC—total antioxidant capacity; hs-CRP—high-sensitivity C-reactive protein; OS—oxidative stress; NO—nitric oxide; SHBG—sex hormone-binding globulin; RCT—randomized controlled trial.

**Table 3:** Cross-domain synthesis of clinical evidence for antioxidant interventions in PCOS.

Outcome Domain	Predominant Evidence Pattern	Relative Evidentiary Strength	Principal Interpretive Constraints
insulin sensitivity/glycemic regulation	improvements reported for selected interventions, primarily in surrogate indices	moderate (context-dependent)	effects modest and phenotype-dependent; limited durability; reliance on surrogate measures
lipid metabolism	no consistent pattern across interventions	low–moderate	baseline dyslipidemia often absent; endpoints variably reported and frequently underpowered
hormonal/endocrine parameters	inconsistent and weak responsiveness	low	endpoints commonly secondary; effects context-specific and poorly reproducible
reproductive outcomes	insufficient and comparator-dependent evidence	low	underpowered trials; frequent co-interventions; sparse live-birth data
oxidative stress biomarkers	biochemical responsiveness commonly observed	moderate (mechanistic level)	poor concordance with clinical outcomes; substantial methodological heterogeneity

Evidence summarized in this table is derived from randomized controlled trials and meta-analyses discussed in Section 4.2, Section 4.3, Section 4.4, Section 4.5 and Section 4.6.

## Data Availability

No new data were created or analyzed in this study. Data sharing is not applicable to this article.

## Abbreviations

The following abbreviations are used in this manuscript:

PCOS	polycystic ovary syndrome
OS	oxidative stress
ROS	reactive oxygen species
ATP	adenosine triphosphate
NF-κB	nuclear factor-κB
NO	nitric oxide
Se	selenium
Cu	copper
Zn	zinc
NADPH	nicotinamide adenine dinucleotide phosphate
RCTs	randomized controlled trials
TC	total cholesterol
LDL-C	low-density lipoprotein cholesterol
HDL-C	high-density lipoprotein cholesterol
TG	triglycerides
VLDL	very-low-density lipoprotein cholesterol
BMI	body mass index
HOMA-IR	Homeostasis Model Assessment of Insulin Resistance
GLUT-4	glucose transporter type 4
AMPK	adenosine monophosphate-activated protein kinase
FSH	follicle-stimulating hormone
LH	luteinizing hormone
MDA	malondialdehyde
TAC	total antioxidant capacity
hs-CRP	high-sensitivity C-reactive protein
SHBG	sex hormone-binding globulin
NAD^+^	nicotinamide adenine dinucleotide
